# Exploring the Immunomodulatory Moonlighting Activities of Acute Phase Proteins for Tolerogenic Dendritic Cell Generation

**DOI:** 10.3389/fimmu.2018.00892

**Published:** 2018-04-30

**Authors:** Inmaculada Serrano, Ana Luque, Josep M. Aran

**Affiliations:** Immune-Inflammatory Processes and Gene Therapeutics Group, Institut d’Investigació Biomèdica de Bellvitge (IDIBELL), L’Hospitalet de Llobregat, Barcelona, Spain

**Keywords:** acute phase proteins, inflammation, monocyte-derived dendritic cells, tolerance, immunotherapy

## Abstract

The acute phase response is generated by an overwhelming immune-inflammatory process against infection or tissue damage, and represents the initial response of the organism in an attempt to return to homeostasis. It is mediated by acute phase proteins (APPs), an assortment of highly conserved plasma reactants of seemingly different functions that, however, share a common protective role from injury. Recent studies have suggested a crosstalk between several APPs and the mononuclear phagocyte system (MPS) in the resolution of inflammation, to restore tissue integrity and function. In fact, monocyte-derived dendritic cells (Mo-DCs), an integral component of the MPS, play a fundamental role both in the regulation of antigen-specific adaptive responses and in the development of immunologic memory and tolerance, particularly in inflammatory settings. Due to their high plasticity, Mo-DCs can be modeled *in vitro* toward a tolerogenic phenotype for the treatment of aberrant immune-inflammatory conditions such as autoimmune diseases and allotransplantation, with the phenotypic outcome of these cells depending on the immunomodulatory agent employed. Yet, recent immunotherapy trials have emphasized the drawbacks and challenges facing tolerogenic Mo-DC generation for clinical use, such as reduced therapeutic efficacy and limited *in vivo* stability of the tolerogenic activity. In this review, we will underline the potential relevance and advantages of APPs for tolerogenic DC production with respect to currently employed immunomodulatory/immunosuppressant compounds. A further understanding of the mechanisms of action underlying the moonlighting immunomodulatory activities exhibited by several APPs over DCs could lead to more efficacious, safe, and stable protocols for precision tolerogenic immunotherapy.

## Introduction

In the superior organisms, inflammation is considered as an evolutionarily conserved, physiological response of the vascularized tissue against external physical, chemical, and biological insults, or internal threats such as metabolic stress. This complex, exquisitely fine-tuned and coordinated process is engaged with the final goal of restoring the homeostasis and repair/regenerate the damaged tissues in a relatively short-time window (1). Whether the insult persists, chronic undesirable inflammation ensues and is associated with a variety of pathologies such as autoimmune processes or vascular diseases. Innate immune cells with the capacity for antigen presentation, that is, specialized antigen-presenting cells (APCs) such as monocytes/macrophages and dendritic cells (DCs), are key players in all phases of inflammation (2). Thus, APCs are involved in the initial sensing of noxious agents through recognition of danger-associated molecular patterns (DAMPs), including pathogen-associated molecular patterns (PAMPs), in the amplification of the defense/protection by locally attracting other immune cells through the vasculature and, finally, are essential effector cells in the resolution of inflammation. All these events are orchestrated mainly by DCs, endowed with high plasticity to bridge innate and acquired immune responses within the inflammatory program (3, 4). Local DAMPs/PAMPs detection by pattern recognition molecules (PRMs), notably the toll-like receptor (TLR) family of proteins, in these cells initiates an adaptive immune process leading to the activation and expansion of antigen-specific effector T lymphocytes in the secondary lymphoid organs (5). Conversely, the absence of pro-inflammatory stimuli or engagement of particular immunoreceptors, such as co-inhibitory receptors (PD-L1, PD-L2, B7-H3, ILT3, etc.) or other tyrosine-based inhibitory motif-containing receptors by a variety of signals maintain DCs in an “immature-like” state. These, “immature” DCs are able to elicit generalized or antigen-specific unresponsiveness/tolerance in central lymphoid organs or in the periphery, promoting the further stimulation of T cells (Treg) able to regulate or suppress other T cells (6). Such actions are crucial to maintain or return to immune homeostasis and to prevent autoimmune responses.

Another inherent aspect of the innate immunity elicited in wounded hosts (particularly those severely injured by trauma or microbial infection), in parallel to the advent of the above-described cellular or acquired immune response, is the prompt occurrence of a prominent non-specific immune-inflammatory response involving systemic physiological and metabolic alterations and affecting tissues/organs distant to the injured site, namely, the acute phase response (7). Thus, immunological stress induces a pro-inflammatory cytokine “storm,” diffusing into the circulation and alerting the liver, which in turn reinforces a protective response through coordinated, cytokine-driven transcriptional changes in hepatocytes, leading to the secretion of a variety of molecules that limit tissue injury and participate in host defense, termed acute phase proteins (APPs), such as the prototypical C-reactive protein (CRP), serum amyloid P (SAP), and serum amyloid A (SAA). These proteins have been traditionally explored as diagnostic/prognostic biomarkers reflecting the presence and intensity of inflammation during infection or injury. Indeed, while most APPs have been traditionally viewed as having a pro-inflammatory function, for example, in immune cell recruitment for efficient pathogen clearance (8), more recent studies are suggesting that a variety of APPs, depending on the microenvironment and through molecular mechanisms not yet completely understood, are able to interact directly with mononuclear phagocytes inducing a regulatory phenotype to these cells.

Mirroring the recent success and increasing importance of cellular immunotherapy strategies for cancer, in the last years a substantial effort has been devoted to generate DCs from blood precursors with tolerogenic features for the treatment of autoimmune diseases, allergy, and transplantation. As the first phase I adoptive tolerogenic DC therapy clinical trials are being concluded, preliminary lessons learned include the overall safety of tolerogenic DC administration, although also highlight present limitations regarding its efficacy. Thus, important current challenges to overcome for a more effective therapeutic outcome include the achievement of antigen-specific tolerogenic responses and, particularly, the maintenance of a “stable” tolerogenic phenotype of the infused DCs regardless of the inflammatory microenvironment that they may confront. Therefore, more progress has to be achieved on the thorough characterization, using both in *in vitro* functional readouts and preclinical assays, of tolerogenic DCs generated through alternative immunomodulatory inducers able to increase their clinical performance in immune-inflammatory pathologies.

In this review, we will consider the potential of APPs as novel immunomodulators. We will overview the current knowledge regarding the interaction of relevant APPs with phagocytes, fundamentally monocytes, and monocyte-derived DCs (Mo-DCs), resulting in a bias toward immune tolerance. A better understanding of the crosstalk between the innate and the adaptive immune systems in homeostasis and inflammatory pathology, taking into account the unique roles of both APPs and DCs, may support therapeutic benefits of APP-induced tolerogenic DCs for transplantation and autoimmunity.

## The Acute Phase Response at the Crossroads Between Innate and Adaptive Immunity

The immediate innate body defense against acute illnesses, that is, the acute phase response, features both, hepatic and extra-hepatic overproduction and release, typically within 24–48 h after the initial insult, of a variety of seemingly biochemically and functionally unrelated APPs into the circulation. In fact, phagocyte sentinels (macrophages, DCs, and neutrophils) sensing eminently damaged, stressed or infected cells, elicit a local pro-inflammatory response, and seek further help by secreting pro-inflammatory cytokines such as IL-6, IL-1, IL-8, TNF-α, and IFN-γ, and releasing a large assortment of “alarmins.” These key mediators travel through the circulation, induce neuroendocrine and behavioral changes (fever, hyponatremia, anorexia, somnolence, and lethargy), and reach the liver, whose most abundant cell type, the hepatocytes, hold also the capability to act as immunological agents and have a central role in the systemic innate immune response through the intravascular secretion of APPs (9). Indeed, APPs conform up to 40 different proteins whose serum concentration increase (positive APPs) or decrease (negative APPs) at least 25% in response to inflammation (10). Positive APPs include soluble PRMs [CRP, SAP, SAA, lipopolysaccharide binding protein, complement components, and α1 acid glycoprotein (AAG)], hemostasis factors (fibrinogen, plasminogen, prothrombin, and plasminogen activators), binding/transport proteins [haptoglobin (Hp), hemopexin, and ceruloplasmin], and antiproteases [α1-antichymotrypsin (AAC), antithrombin (AT), α1-antitrypsin (AAT), and α2-macroglobulin (α_2_M)]. These proteins participate in host defense (e.g., attracting inflammatory cells, inactivating proteolytic enzymes, activating complement, opsonizing, and clearing infectious agents) and limit tissue injury (scavenging free radicals and modulating the host’s immune response). Conversely, negative APPs comprise albumin, AT, transferrin, transthyretin, transcortin, and retinol-binding protein (8). It has been suggested that reduced albumin production enhances the amino acids “pool” available for positive APP production, and that decreased transferrin production could protect the host by starving microorganisms of the iron required for growth and virulence expression (11).

Based on their degree of response to inflammatory stimuli, APPs can be grouped as strong (more than 100-fold increase in blood levels; CRP, α_2_M, SAA), moderate (2–10-fold increase; haptoglobulin, fibrinogen, AAT), or weak (up to twofold increase; C3, ceruloplasmin). While strong APPs usually increase abruptly within the first 24–48 h after an acute inflammatory event, and further experience a quick decline related to their relatively short half-life, moderate to weak APPs are more likely present during chronic inflammatory processes. According to the differential regulation of their synthesis by cytokines, positive APPs can also be classified in type I and type II. Type I are induced by IL-1-like pro-inflammatory cytokines (SAA, CRP, C3, AAG, and SAP), and type II are induced by IL-6-like cytokines (fibrinogen, Hp, AAC, AAT, and α_2_M). In turn, the production of hepatic APPs may also be influenced by other cytokines and by hormones (insulin, dexamethasone, glucagon, and/or epinephrine) (12). Thus, at the level of the organism, the complex neuroendocrine-immunological axis seems to efficiently modulate the acute phase response through various feedback loops (13). For instance, cytokines released from monocytes/macrophages activated locally through noxious inflammatory agents stimulate the brain to release stress-response neuropeptides such as corticotropin (ACTH), which acts into the adrenal glands inducing glucocorticoid production. Glucocorticoids can downregulate pro-inflammatory cytokines (IL-1, TNF-α).

Due to their stability in the circulation compared with cytokines, which are cleared from the circulation within a few hours, several APPs have been extensively used as diagnostic/prognostic biomarkers because their increased/decreased levels reflect the presence and intensity of inflammation during infection or injury, remaining unchanged for 48 h or longer. Nevertheless, although presenting high sensitivity, the diagnostic value of APPs is being questioned due to their low specificity (14).

## Inflammatory DCs in Inflammation

Relevant features of the acute phase response are an increase in the number of peripheral leukocytes and the dilation and leakage of the vasculature through the release of inflammatory mediators such as reactive oxygen species, arachidonate metabolites, and pro-inflammatory cytokines and chemokines (15). Pro-inflammatory cytokines activate and mobilize blood cell precursors in both bone marrow and peripheral blood (16–18). Moreover, stimulated endothelial cells allow the extravasation and migration of circulating leukocytes. Among these, Mo-DCs have been appealing due to: (1) their influence on adaptive immune function and rapid accumulation in the inflammatory focus and (2) their easy *ex vivo* isolation, amplification, and manipulation. Mo-DCs arise from monocyte precursors both *in vitro* and *in vivo* (19, 20). Monocytes are recruited to sites of inflammation, having a major role in the protective immune response of the host (21). For instance, local differentiation of monocytes into inflammatory macrophages and DCs is induced in response to natural killer cell-produced IFN-γ (22). In fact, by depletion of tissue-resident cell populations it has been shown that circulating monocyte precursors in the blood can replenish functionally specialized macrophages and DCs (23), which reinforces the concept of blood monocytes as reservoirs that can be utilized on demand, particularly in inflammatory processes where monocyte recruitment is strongly increased. Accordingly, monocytes have been shown to migrate to inflammatory sites and differentiate into DCs in various murine models of inflammation (24, 25). Sequential trafficking and/or differentiation of the different monocyte subsets to the sites of inflammation is likely modulated by diverse mechanisms (26–28). Following tissue damage, classical monocytes (human: CD14^++^CD16^−^; mouse: Ly6C^+^CCR2^high^CX_3_CR1^low^) appear to be recruited within the first few hours, after their egression from the bone marrow being modulated by the CCR2–CCL2/CCL7 axis (29). Once in the inflammatory milieu, they differentiate into DCs and macrophages and exert a potent pro-inflammatory immune response through high-level production of IL-1β and TNF-α, among other protective functions (30–32). When the progression of the immune-inflammatory response is not halted, the prolonged action of classical inflammatory monocytes may result in tissue damage and drive autoimmunity (33). Several days after the initial damaging insult, acute inflammation enters in a resolution phase where the classical monocyte levels are reduced and progressively replaced by intermediate [CD14^+(+)^CD16^+^] and non-classical (human: CD14^+^CD16^++^; mouse: Ly6C^−^CCR2^low^CX_3_CR1^high^) monocytes, which relay on the CX_3_CR1–CX_3_CL1 axis to accumulate in the damaged tissue and, after DC/macrophage differentiation, secrete anti-inflammatory cytokines (IL-10, TGF-β) that counteract tissue injury and promote wound healing (34). Certainly, it has been suggested that, in response to inflammatory stimuli, patrolling non-classical CD16-expressing monocytes could leave the blood vessels and function as DC precursors (35). Thus, these inflammatory Mo-DCs seem to hold unique features influenced by the microenvironmental status of the inflamed tissue, boosting more potent immune responses DCs derived from classical monocytes, and better immune tolerance DCs generated from non-classical monocytes (36).

Monocytes from human or mouse peripheral blood or bone marrow are widely utilized to generate *in vitro* large amounts of Mo-DCs upon differentiation, typically with IL-4 and GM-CSF (37), allowing comprehensive mechanistic studies regarding their key role in the immune-inflammatory processes at the molecular level and to initiate DC therapy approaches in the clinic. In fact, a comparative transcriptional profiling has revealed that human DCs isolated from inflammatory fluids are the *in vivo* counterpart of *in vitro*-generated Mo-DCs from CD14^+^ monocytes (38), in the same way that murine inflammatory DCs share equivalent developmental and functional features to *in vitro* GM-CSF/IL-4-induced BM-DCs (39).

Monocyte-derived cells have been deemed essential for inducing protective Th1 cell-mediated immunity following both pathogen infection and non-infectious conditions (40, 41), and may acquire DC-specific functions such as cross-presentation (41, 42).

Conversely, DCs play a key role in tolerance, whether participating in the negative selection of autoreactive T cells in the thymus (central tolerance) (43), or limiting effector T cells through deletion or anergy and, instead, promoting Treg differentiation (peripheral tolerance). A variety of mechanisms are orchestrated by DCs to induce tolerance and suppress inflammatory responses against innocuous stimuli, including the overexpression of inhibitory immunoreceptors (e.g., PD-L1, B7H, and CD80/86), the ligand-activated transcription factor aryl hydrocarbon receptor, the pore-forming cytolytic protein perforin, and the release and/or control of several immunomodulatory mediators, such as anti-inflammatory cytokines (IL-10, IL-27, and TGF-β), indoleamine 2,3-dioxygenase (IDO) metabolites, retinoic acid, vitamins A and D, ATP, and adenosine [see Ref. (44, 45), and references therein]. The regulatory function of DCs is determined by their maturation/activation status (46). Hence, tolerogenic DCs hold an “immature” or “semi-mature” state.

A myriad of recent studies has reported the *in vitro* generation of monocyte-derived “permissive,” “tolerogenic,” “regulatory,” “alternatively activated,” or “maturation-resistant” cell types (47), although most attention has been focused on Mo-DCs. This is being achieved by incubation with a variety of different biological or pharmacological agents such as cytokines (IL-10, TNF-α, IFN-γ, TGF-β, IL-21, and thymic stromal lymphopoietin), immunosuppressant drugs (dexamethasone, tacrolimus, and mycophenolate), organic molecules (vitamin D_3_, salycilate, vasoactive intestinal peptide, intravenous immunoglobulin, and hepatocyte growth factor), other agents (pathogen products, mesenchymal stem cells), or their combinations, or by genetic engineering (48–50). Mimicking the *in vivo* circumstances, the resulting tolerogenic Mo-DCs are characterized essentially by reduced surface expression of co-stimulatory molecules (CD80, CD86, and CD40) and maturation markers (CD83), increased expression of inhibitory receptors (ILT3, PD-L1, and PD-L2), reduced or null production of pro-inflammatory cytokines (IL-12, TNF-α, IFN-γ, and IL-8) and, conversely, increased production of anti-inflammatory cytokines (IL-10, TGF-β), even in the presence of inflammation (51–54). Thus, the main features of these cells would be to present a state of unresponsiveness through hampering key activation/maturation pathways such as the pro-inflammatory NF-κB pathway, and to support the differentiation and maintenance of different types of Treg cells.

## Tolerogenic Actions of APPs on DCs

There are clear evidences showing that the acute phase response can directly influence the differentiation of DCs toward a tolerogenic state. In sepsis, an overwhelming systemic inflammatory response syndrome, an expansion of intermediate monocytes has been detected in the circulation (55). Monocytes from sepsis patients preferentially differentiated into alternative CD1a^−^ DCs, holding increased capacity to induce Foxp3^+^ Treg cells, when compared with monocytes from healthy individuals in which classical monocytes predominated (56). On the other hand, the hepatic APPs SAA and Cxcl1/KC cooperatively promoted myeloid-derived suppressor cell (MDSC) mobilization, accumulation and survival, reversed dysregulated inflammation, and restored survival of mice deficient for gp130 (the signaling receptor shared by IL-6 family cytokines) undergoing polymicrobial sepsis (57). Thus, hepatocytes may also modulate innate immune cells through the acute phase response, for example, by recruitment and promotion of MDSC function.

**Figure 1:**
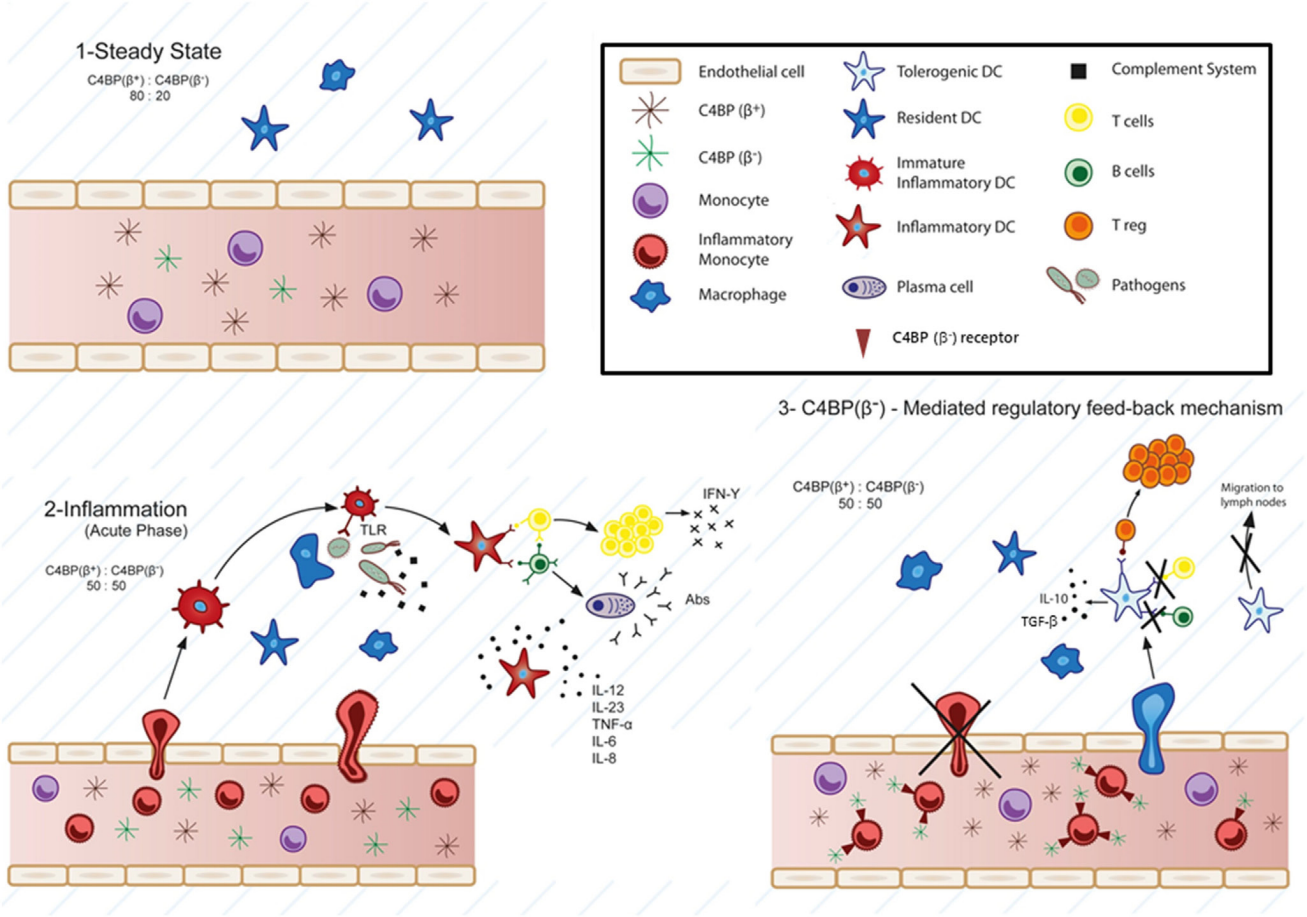
Physiological involvement of C4b-binding protein (C4BP)(β−) in cell-mediated immune-inflammatory responses. According to this model, at the steady-state, the classical and lectin pathways complement inhibitor C4BP circulates in two main isoforms: C4BP(β+) (80%) and C4BP(β−) (20%) (1). Strong pro-inflammatory conditions (acute phase) induce a modification of the C4BP(β+):C4BP(β−) ratio to 50:50, through increased levels of the acute phase protein C4BP(β−) (2). Several evidences support a specific action of overexpressed C4BP(β−) over inflammatory monocytes and monocyte-derived dendritic cells (DCs). Strong inflammatory stimuli (infection, lupus nephritis, etc.) trigger the presence of inflammatory monocytes in the blood, which are actively recruited to inflamed tissues, and differentiated to inflammatory DCs, having the ability to stimulate naïve T cells. Under these conditions, besides its function as complement inhibitor, the increased presence of C4BP(β−) in the blood would act in one or both ways upon engaging one or some, as yet unknown, cell surface receptor(s): 1) reducing transendothelial migration and accumulation of the inflammatory monocytes into the inflamed tissue and 2) inducing a tolerogenic phenotype in the recruited inflammatory DCs, which would led to: (a) inhibition of T cell proliferation and differentiation into Th1, Th2, and/or Th17 cells depending on the inflammatory microenvironment, (b) decreased pro-inflammatory cytokine secretion (IL-12, TNF-α, IFN-γ, etc.), (c) reduced migration to the lymph nodes, and conversely, to: induction of anti-inflammatory cytokine release (IL-10, TGF-β, etc.), and (d) Treg generation within the inflamed tissues (3).

Accordingly, it is not unreasonable to consider a number of APPs, acting either systemically or locally in a restricted time window coinciding with a parallel increase of monocyte recruitment toward the inflammatory focus, as a part of a protective network to restrain the harmful consequences of continued overinflammation. That is, APPs could directly exert a feedback loop redirecting the differentiation of these inflammatory monocytes to regulatory or tolerogenic DCs, in an attempt to regain homeostasis and maintain tissue integrity through the resolution of the immune-inflammatory response (Figure 1). We will now focus in representative APPs and APP-related proteins that are able to induce tolerogenesis through modulation of Mo-DC differentiation and/or maturation.

Soluble PRMs are a heterogeneous group of molecules (collectins, ficolins, pentraxins, and other complement components) belonging to the humoral arm of innate immunity that have been proposed to represent the functional ancestor of antibodies (58). They share basic functions with the membrane-bound PRMs from DCs, such as the recognition of “non-self” and “modified self” and, additionally, play an important role in opsonization and complement activation. In the last years, several studies have evidenced that APPs, particularly soluble PRMs, acting directly in the early stages of monocyte differentiation mediated by GM-CSF/IL-4 (a faithful *in vitro* model for the generation of inflammatory DCs), are able to confer a tolerogenic phenotype and function to the ensuing Mo-DCs, although the detailed molecular mechanisms of APPs action over DCs are still not known for most of them.

In the next paragraphs, we will address the state of understanding and arguments regarding APP-mediated tolerogenic DC generation and functional outcome, according to common features currently defining tolerogenic DCs.

### Pentraxins

Pentraxins constitute a superfamily of evolutionarily conserved multimeric and multifunctional proteins sharing an 8-amino acid “pentraxin domain” (HxCxS/TWxS, where “x” is any amino acid) in their carboxy terminus. Based on the primary structure of the promoter, pentraxins are divided into short pentraxins (CRP and SAP) and long pentraxins (PTX3) (58). Both CRP and SAP are homooligomeric proteins arranged in a ~25 kDa subunit pentameric radial symmetry and hold 51% amino acid sequence identity. They constitute the main APPs in human and mouse, respectively, are produced by hepatocytes and have wide capacity for pathogen recognition, phagocytosis, and cytokine secretion through interaction with Fcγ receptors (59). Moreover, CRP and SAP are able to regulate the activation of the complement system by interaction with C1q, ficolins, C4b-binding protein (C4BP) and factor H, favoring efferocytosis and preventing the onset of autoimmune diseases (60, 61).

C-reactive protein has been shown to transform biological functions of Mo-DCs toward a tolerogenic phenotype. Interestingly, when CRP was added at the early stage of Mo-DC differentiation from CD14^+^ monocytes, it downregulated surface expression of DC-SIGN and the antigen uptake molecules CD205 and CD206, resulting in reduced endocytosis capacity (62, 63). Moreover, LPS-mediated Mo-DC maturation was also impaired, through downregulation of co-stimulatory molecules CD80 and CD86, and of the maturation marker CD83, inhibition of allogeneic T cell proliferation and decreased production of pro-inflammatory cytokines (IL-12, IL-8, IL-6, TNF-α, MIP-1α, MIP-1β, and MCP-1). These effects seemed to be mediated through the immunoreceptor FcγRII/CD32, which is downregulated during differentiation into Mo-DCs. Conversely, another study reported just the opposite, that is, CRP was able to activate Mo-DCs through upregulation of DC activation markers (CD40, CD80, CD83, and CCR7) and induced allogeneic T cell proliferation and IFN-γ production (64). Nevertheless, in that case the pulsation of Mo-DCs was started at day 6 of culture, once the Mo-DCs were fully differentiated. These results evidence the restricted tolerogenic activity window characterizing CRP, at the initial steps of Mo-DC differentiation. Analogously, human SAP has been reported to bind strongly to monocytes but weakly to differentiated Mo-DCs (65). SAP also inhibits neutrophil recruitment and monocyte to fibrocyte differentiation, in part, by binding to the FcγRs (66, 67), and polarizes macrophages toward an immunoregulatory phenotype through PI3K/Akt-ERK signaling (68). Thus, SAP regulates key components of the innate immune system and inflammation.

Pentraxins is a multimeric 340 kDa glycoprotein with a complex quaternary structure (elongated, with a large and a small domain interconnected by a stalk region) composed of two tetramers linked by interchain bridges to form an octamer. PTX3 expression is induced in a variety of cell types (particularly in phagocytes) by inflammatory cytokines, TLR agonists or pathogens, binds to a wide range of microorganisms, and plays a relevant role in host defense and inflammation (69), for example, by regulating leukocyte recruitment (70). Moreover, analogously to CRP and SAP, PTX3 is also able to modulate the activation of the complement system by binding C1q, ficolins, mannose-binding lectin (MBL), and the complement regulators C4BP and FH, and increases phagocytosis in an FcγRII-dependent manner. Hence, PTX3 binds to apoptotic cells and recruits C4BP, limiting complement activation and an exacerbated inflammatory response (71). In this context, PTX3 reduces the release of TNF-α and IL-10 by LPS-challenged Mo-DCs, and consistently inhibits the upregulation of membrane molecules (CD86, HLA-ABC, HLA-DR) on an inflammatory cell surface induced by LPS. Moreover, PTX3 also induces macrophages to secrete anti-inflammatory cytokines such as TGF-β and IL-10 (72), modulates LPS-induced inflammatory response and attenuates liver injury (73).

### Complement Components

The evolutionarily conserved complement system, in addition to its crucial function in the innate defense against common pathogens, holds also a key regulatory non-immunogenic role in the “silent” clearance of immune complexes from the circulation and apoptotic cells from damaged tissues, in close crosstalk with the mononuclear phagocyte system (74). We have recently discussed the “non-canonical” activities of a variety of complement effectors and modulators able to transform DCs toward a tolerogenic phenotype (75). Thus, we will instead focus here on the functional outcome of a few representative complement components directly interacting with Mo-DCs.

In addition to their central role as complement cascade initiators for microbial phagocytosis and killing, it is becoming evident that both, complement cascade initiators such as mannose-binding lectin (MBL) and soluble complement inhibitors such as C4BP, are able to promote an immunomodulatory and anti-inflammatory environment by direct interaction with DCs and other immune cells.

MBL, the prototypic initiator of the lectin pathway of complement activation, belongs to the collectin family and, through its carbohydrate-recognition domains, is able to bind to oligosaccharides (mannose, *N*-acetyl-glucosamine) on the pathogen surface (76). DCs from MBL-deficient individuals showed increased IL-6 production and poor allogeneic T cell responses, features of pathogen-stimulated DCs, which could be reversed by *in vitro* addition of MBL (77). In fact MBL, at supraphysiological concentrations, influences the phenotype and function of DCs by attenuating LPS binding to immature DCs and their further maturation and pro-inflammatory cytokine production (IL-12, TNF-α), while preventing allogeneic T lymphocyte proliferation (78). Moreover, MBL not only attenuates LPS-induced Mo-DC maturation, but also affects early Mo-DC differentiation from CD14^+^ monocytes, yielding Mo-DCs with tolerogenic features (low MHC-II, CD80 and CD40 expression, increased IL-10 and IL-6 secretion, and reduced T cell alloproliferation), and being possibly mediated by members of the STAT family (79).

Among the complement inhibitors, the regulator of the classical and lectin pathways of complement activation C4BP has a complex oligomeric structure. The major C4BP isoform, C4BP(β+), has an heterooligomeric radial structure (570 kDa). It is composed by seven identical 70 kDa modular α-chains (responsible for the complement inhibitory activity, and for pentraxin, heparin, DNA, and pathogen binding, among others), and a single 40 kDa β-chain (high-affinity binding site for anticoagulant vitamin K-dependent Protein S, allowing a strong interaction with apoptotic/necrotic cells) (80, 81). The minor C4BP isoform, C4BP α7β0 or C4BP(β−), holds the same oligomeric structure and complement inhibitory function than C4BP(β+), but lacks the β-chain. Under acute phase conditions (poly-traumatisms, sepsis) the levels of circulating C4BP(β−) isoform increase significantly as a consequence of the differential hepatic regulation of the α- and β-chains by pro-inflammatory cytokines (82). Thus, C4BP(β−) is a genuine APP. We have shown that the C4BP(β−) isoform, but not the C4BP(β+) isoform, by direct interaction with Mo-DCs through as yet unknown receptor(s), only in the early stages of monocyte to Mo-DC differentiation, is able to confer an anti-inflammatory, tolerogenic phenotype to these cells, retaining a high-endocytic activity, and morphological features of immaturity. Upon LPS priming, these C4BP(β−)-treated Mo-DCs featured low-surface expression of CD83, CD80, and CD86, inhibition of pro-inflammatory IL-12, TNF-α, IFN-γ, IL-6, and IL-8 production and, instead, increased expression of anti-inflammatory IL-10 and TGF-β, reduced CCR7 expression and chemotaxis, and promoted Treg expansion. Moreover, C4BP(β−) induced tolerogenic DCs with increased viability and yield when compared with the immunomodulator vitamin D3, and similarly prevented T cell alloproliferation (83).

Although perhaps not a *bona fide* APP, C1q, the recognition unit from the classical pathway of complement activation and major component of the C1 complex, binds to various APPs including CRP, SAP, and PTX3, thereby regulating the classical complement pathway. C1q has also been recognized to modulate cellular functions within the adaptive immune response (84). Certainly, C1q has even been proposed as a tolerogenic DC marker because relevant immunomodulatory agents such as dexamethasone, IL-10, or vitamin D3 are able to induce at least a 10-fold overexpression of C1q at both mRNA and protein levels in Mo-DCs (85). The regulatory effects of C1q on monocyte/DC precursors could be mediated by gC1qR, occurring within a narrow timeframe of monocyte to Mo-DC transition and being influenced by the microenvironment. Accordingly, while in the presence of danger signals C1q would recognize and bind antigens through its globular head domains, leading to activation of a pro-inflammatory immune response in immature Mo-DCs, in the absence of danger signals C1q would maintain immature Mo-DCs in a tolerance state through gC1qR (86). Certainly, gC1qR ligation on the surface of Mo-DCs suppresses TLR4-induced IL-12 production through PI3K pathway activation (87). Furthermore, an alternative mechanism of C1q-mediated immunomodulation involves high-affinity binding between C1q and the inhibitory immunoreceptor LAIR-1, which inhibits monocyte-to-Mo-DC differentiation (88). More recently, this interaction has been refined through the characterization of a tri-molecular engagement encompassing C1q-CD33/LAIR-1 crosslinking (89).

### Hemoglobin- and Iron-Binding Proteins

Essential cellular processes, such as energy generation, DNA replication, oxygen transport, and protection from oxidative stress are dependent on iron. Since bacterial pathogens also require iron for replication and infection, iron sequestration strategies from vertebrates constitutes a significant form of nutritional innate immunity (90). Thus, in homeostatic healthy conditions iron is largely intracellular and sequestered within ferritin. Conversely, acute inflammatory processes such as infection, include the release of lactoferrin from secondary granules contained within polymorphonuclear leukocytes. Furthermore, hemoglobin released by physiological and pathological hemolysis is captured by Haptoglobin (Hp). All together, these proteins ensure a virtually free iron environment in vertebrate tissues.

Ferritin is a major tissue iron-binding protein with a molecular weight of 500 kDa, whose main function is to store iron in a soluble non-toxic form, protecting the cell from iron-mediated redox reactions. The levels of this APP remain elevated in many chronic inflammatory diseases such as periodontitis (91). Ferritin is composed of 24 subunits consisting of heavy (H) and light (L) chains, and may heterooligomerize forming isoferritins depending on the proportions of H and L chains (92). The immunosuppressive effects of cancer cell supernatants, such as melanoma supernatants, correlated with their content of H-ferritin. Accordingly, H-ferritin has been shown to inhibit anti-CD3-stimulated lymphocyte proliferation, probably mediated by increased IL-10 production (93). Importantly, H-ferritin is also able to induce a semi-mature, tolerogenic phenotype on Mo-DCs featuring increased expression of CD86 (B7-2) and B7-H1, and the activation of IL-10-producing Treg cells (94).

Lactoferrin, also known as lactotransferrin, is produced in a number of tissues and is frequently found in mucosal secretions and neutrophil secretory granules (95). This 80 kDa iron-binding glycoprotein is an important component of innate immunity, and holds a key role in the protection of mucosal surfaces from microbial infections (96). Lactoferrin also modulates innate and adaptive immune-inflammatory responses, including cytokine production, promotion of T and B cell maturation, and enhancement of delayed-type hypersensitivity against defined antigens. Moreover, it has been suggested that lactoferrin might exert adjuvant activity, enhancing DC function to promote generation of antigen-specific T cells (97). In contrast, bovine lactoferrin (bLF) seems to play an opposite role. Thus, Mo-DCs differentiated in the presence of bLF showed a fully tolerogenic or immunomodulatory behavior [potent anti-inflammatory activity, high-endocytic capacity, increased expression of molecules with negative immunoregulatory functions (ILT3, PD-L1, IDO, and SOCS3), CCL1 production, and impaired capacity to undergo activation and to promote Th1 responses]. bLF is internalized and seems to reach the nucleus, although the molecular details mediating the bLF-mediated transcriptional regulation of Mo-DC differentiation are still unknown (98, 99).

Hp is the major hemoglobin-binding protein in plasma. This APP, whose hepatic expression is induced by inflammatory mediators such as IL-6-type cytokines, interacts with free hemoglobin neutralizing and restricting its oxidative damage to various organs (100). Hp has been suggested to exert immunomodulatory effects constituent with suppression of lymphocyte function (101). During physiological and pathological hemolysis, the Hp-CD163-heme oxygenase (HO-1) pathway efficiently scavenges and circumvents hemoglobin/heme-induced toxicity. This pathway plays an anti-inflammatory role in phagocytes, and the resulting heme metabolites, such as bilirubin, reinforce its cytoprotective and anti-inflammatory efficacy (102). Hp seems also to prevent epidermal Langerhans cells from spontaneously undergoing functional maturation in the skin, inhibiting their capacity to activate autologous T cells *in vitro* (103).

### Other APPs and APP-Related Proteins

Serum amyloid A is an APP produced mainly by the hepatocytes, but also by other cell types such as macrophages, smooth muscle cells, chondrocytes, epithelial cells, and adipocytes, under pro-inflammatory stimuli (58). SAA interacts with Gram-negative bacteria and, through its opsonic activity, increases their phagocytosis and the production of TNF-α and IL-10 by phagocytes (104). SAA has also been recently shown to be involved in the expression of the “alarmin” IL-33 by monocytes and macrophages (105). Notably, it has been recently shown that SAA-stimulated monocytes (HLA-DR^hi^ HVEM^lo^) most resemble immature Mo-DCs, and are able to drive Treg proliferation (106). SAA is also a chemoattractant for immature Mo-DCs through formyl peptide receptor like 1/formyl peptide receptor 2 (107). Furthermore, mice lacking SAA3, an acutely expressed isoform found in non-primate mammals, develop metabolic dysfunction, and exacerbated pro-inflammatory responses from innate immune cells. Particularly, bone marrow-derived DCs from SAA3^(−/−)^ mice produce increased levels of IL-1β, IL-6, IL-23, and TNF-α in response to LPS compared with cells from wild-type mice (108). Thus, endogenous SAA3 likely modulates metabolic and immune homeostasis.

α1-Antitrypsin, a member of the SERPIN superfamily of protease inhibitors, is a major inhibitor of the neutrophil-derived serine proteases [neutrophil elastase (NE), cathepsin G, and proteinase 3]. It has a primary anti-inflammatory role by irreversible binding and inactivation of NE, protecting the lung against the destructive effects of NE released by degranulating neutrophils during inflammation (109). AAT is predominantly produced by the liver, and its secretion, increased under acute phase conditions, is mediated by pro-inflammatory cytokines (IL-6, IL-1β, and TNF-α) (12). Recent studies have reported tolerogenic activities of AAT that are difficult to explain solely by serine-protease inhibition or by its anti-inflammatory actions (110). Circulating AAT is bound to lipoprotein particles (LDL and HDL) and docks onto lipid-rafts. Thus, TLR2 and TLR4 contained in lipid-rafts from macrophages and DCs are downregulated by AAT (111). Moreover, AAT induces a tolerogenic phenotype on DCs characterized by low levels of CD40, CD86, and MHC class II, increased production of IL-10 and enhanced generation of Tregs through a so far unknown mechanism. These tolerogenic DCs maintain, nevertheless, the inflammation-driven cell migration capacity (112). In fact, AAT monotherapy has been shown to induce tolerance in islet allograft and kidney transplantation (113, 114), graft-versus-host disease (115), improved islet function in type I diabetes (116), and attenuated lupus nephritis (117).

Fibrinogen is synthesized mainly by hepatocytes, and its level increases substantially during infections and inflammatory conditions. This 340 kDa glycoprotein, made up of two identical subunits joined together by disulfide bonds, functions as a blood coagulation factor, supporting platelet aggregation, and fibrin cloth formation at the site of vessel injury. Fibrinogen had an Mo-DC maturation effect comparable with poly I:C, TNF-α, and PGE_2_, but it failed to induce IL-12 production (118). On the other hand, it has been recently reported that fibrinogen cleavage products generated by protease allergens, through induction of IL-13 production by mast cells, increased the number of T_H_2-favorable (PD-L2^+^) DCs in allergic asthma (119). Interestingly, another member of the fibrinogen-related protein superfamily, soluble fibroleukin or fibrinogen-like protein 2 (sFGL2), highly inducible by IFN-γ and with features of APP, has a 50 kDa weight and is highly expressed in cytotoxic T cells and Tregs upon activation (120). sFGL2 seems to act as an immunosuppressor, repressing the proliferation of alloreactive T lymphocytes and the maturation of DCs (121, 122). Thus, by binding to FcγRIIB and FcγRIII, sFGL2 can adjust the antigen presentation ability of APCs. Accordingly, the levels of Th2 cytokines and the activity of DCs have found to be increased in FGL2-deficient mice (123).

## Immunotherapeutic Potential of APPs for Tolerogenic DC Induction

Pharmacological immunosuppression has gone mainstream of past and, still, current therapeutic strategies to prevent transplant rejection and to restore autoantigen tolerance in autoimmune disorders. Yet, the downside of the immunosuppressive regimens is the appearance of numerous and often severe side effects and increased risk of infection as a consequence of the general suppression of the host immune system (124–126). Thus, the attractive concept of using DCs, central orchestrators of other immune cells, with the aim to modulate immune-inflammatory responses that have gone awry while leaving protective immunity intact is becoming gradually a reality in the clinical setting. In fact, in addition to being explored in experimental animal models of autoimmune diseases such as collagen-induced arthritis (127, 128), diabetes (129, 130), and experimental autoimmune encephalomyelitis (EAE) (131), along with experimental graft rejection after transplantation (132, 133), tolerogenic DCs have recently been, and are currently being tested in phase I clinical trials for alloimmune (transplantation, graft-versus-host disease) and autoimmune processes (type I diabetes, rheumatoid arthritis, multiple sclerosis, and Crohn’s disease), and allergy, and there are ongoing collaborative efforts to harmonize/standardize tolerance-inducing therapies for upcoming trials (134–136).

The identification, characterization and, most notably, isolation and amplification of genuine regulatory or tolerogenic DCs populating a given healthy or diseased tissue, much like MDSCs, has proven a daunting task and a real limitation when planning to adoptively transfer them for therapeutic benefit. Therefore, due to the high plasticity of mononuclear myeloid cells such as monocytes, well-established *ex vivo* protocols of monocyte to Mo-DC expansion and differentiation, relying in the use of inflammatory cytokines (GM-CSF and IL-4) have become instrumental to adopt Mo-DCs as therapeutic cell products for clinical use (137).

A central aspect for the successful clinical application of tolerogenic Mo-DC relates to their development and manufacture. As previously stated, a variety of agents have been employed *in vitro* to skew Mo-DCs toward a tolerogenic or regulatory phenotype (notably vitamin D3, immunosuppressive drugs-like dexamethasone, or NF-κB inhibitors), opposing their “natural” tendency to be activated *in vivo* in a pro-inflammatory environment. Yet limited efficacy has been reported in terms of disease outcome, although most trials have noted an increase in Treg levels in the recipient’s blood during tolerogenic Mo-DC administration (138–140). Clearly, maintaining tolerogenic Mo-DCs in an activation- or maturation-resistant state is a fundamental requirement for a successful tolerogenic Mo-DC therapy, because unstable tolerogenic Mo-DCs able to reverse back *in vivo* to an immunogenic phenotype in contact with a pro-inflammatory microenvironment could aggravate the pathology. Indeed, semi-mature DCs, considered tolerogenic in *in vitro* assays, may become immunogenic when administered *in vivo* (141, 142). Furthermore, it has been recently reported that continuous treatment of DCs during their differentiation from bone marrow cells (10-day treatment) with the histone deacetylase inhibitor suberoxylanilide hydroxamic acid generated tolerogenic DCs that, however, were not stable and, therefore, inefficacious when administered in mice with EAE (143). Hence, the possibility to anticipate and modulate the stability of tolerogenic Mo-DCs *in vivo*, particularly in the pro-inflammatory allo- or autoimmune environments in which these cells are applied, would enhance their therapeutic efficacy. As yet, there is not enough mechanistic knowledge to ascertain which stimuli guarantee the induction of stable tolerogenic Mo-DCs adapted to particular *in vivo* situations. Still, in tolerogenic Mo-DC conditioning protocols, establishing the appropriate timing and intensity of the tolerogenic reagent treatment, its toxicity, as well as the migration capacity of the resulting conditioned cells are crucial aspects to take into account for a successful Mo-DC-based immunotherapy (134). For example, both rapamycin- and dexamethasone-conditioned cell cultures have been shown to markedly reduce DC recovery (144, 145). Importantly, none of the APPs proved cytotoxic in the Mo-DC cultures, most likely because of the wide range of physiological concentrations that these proteins are able to reach in serum, fluctuating between homeostatic and acute phase conditions. Thus, APP-derived tolerogenic Mo-DCs might overcome some of the limitations of the current tolerogenic Mo-DCs employed for immunotherapy approaches regarding consistency, safety, and efficacy (124, 146).

On the other hand, the common tolerogenic moonlighting activity of APPs over Mo-DCs seems surprising, given the variety of different physiological functions ascribed to these proteins. Remarkably, nearly all of them share a complex, oligomeric, and multi-modular structure (Table 1), providing flexibility in their capacity for binding, with a different grade of specificity, a variety of humoral and/or cellular determinants, including different receptors in the surface of Mo-DCs. This feature might constitute an advantage for the fine-tuning of the desired tolerogenic phenotype on Mo-DCs.

**Table 1:** Immunomodulatory actions of acute phase proteins (APPs) on dendritic cells (DCs).

APP		Structural information	Canonical function	Tolerogenic activity	Reference
**Soluble pattern recognition molecules**					

	Pentraxins				

	C-reactive protein	Annular, ring-shaped, pentameric protein (~125 kDa)	Activation of the complement system Pathogen protection	CD209↓, CD40↓, CD83↓, CD80↓, CD86↓ Endocytosis↓ IL-12↓, MCP-1↓, TNF-α↓, IL-6↓, IL-8↓, MIP-1α↓, MIP-1β↓ Allogeneic T lymphocyte proliferation↓	(62, 63)

	Serum amyloid P	Annular, ring-shaped, pentameric protein (~125 kDa)	Activation of the complement system Binding to fibrils and amyloid deposits Pathogen protection	IL-12↓, IL-10↑	(65, 66, 68)

	Pentraxin 3	Cyclic multimeric structure. Complex quaternary structure composed of two tetramers linked by interchain bridges to form an octamer (~340 kDa). Proposed stabilized decameric protein (~450 kDa)	Activation of the complement system Pathogen protection	CD86↓, HLA-ABC↓, HLA-DR↓ TNF-α↓, IL-10↑, TGF-β↑	(72, 73)

	Serum amyloid A	Oligomeric apolipoprotein. Probably trimeric (~35 kDa) or hexameric structure (~70 kDa)	Cholesterol transport	HLA-DR↑, HVEM↓ IL-1β↑, IL-6↑ Treg generation↑	(106)

	Complement components				

	Mannose-binding lectin	Oligomer (400–700 kDa). Tetrameric structure build of subunits containing three presumably identical peptide chains	Activation of the lectin pathway of complement	CD40↓, CD80↓ IL-12↓, TNF-α↓, IL-10↑, IL-6↑ Allogeneic T lymphocyte proliferation↓	(78, 79)

	C4b-binding protein (C4BP(beta-))	Oligomeric radial structure composed of seven identical α-chains (~520 kDa)	Inhibition of the classical pathway of complement	CD83↓, CD80↓, CD86↓ IL-12↓, TNF-α↓, IFN-γ↓, IL-6↓, IL-8↓, IL-10↑, TGF-β↑ Th1 proliferation↓ Treg generation↑	(83)

**Antiproteases**					

	α1-antitrypsin	Monomer (52 kDa)	Serine-protease inhibition	CD40↓, CD86↓, MHC-II↓ TNF-α↓, IL-1β↓, IL-12↓, IL-6↓, IL-10↑, CCR7↑ Allogeneic T lymphocyte proliferation↓ Treg generation↑	(112–117)

**Toxin binding/transport**					

	Haptoglobin	Preproprotein processed to yield both α- and β-chains, which combine to form a tetramer (~100 kDa), or polymerize (~900 kDa), depending on its phenotype	Free plasma hemoglobin binding	MHC-I↓, MHC-II↓, B7↓, CD40↓ IL-12↓ Autologous, naive T cell activation↓	(103)

**Hemostasis**					

	Fibrinogen	Composed of three non-identical pairs of disulfide-bonded chains (~340 kDa)	Blood clotting	CD83↑, CD86↑ IL-12↓ Allogeneic T lymphocyte proliferation↑	(118)

**Binding/transport of essentials**					

	Ferritin	Mixture of oligomers. Forms 24-mers (~480 kDa)	Iron storage and transport	CD86↑, B7-H1↑ IL-10-producing Treg generation↑	(94)

**Other APP-related proteins**					

	Fibrinogen-like protein 2	Oligomer consisting of four monomers (~200 kDa). Structure homologous to fibrinogen and tenascin	Membrane-bound FGL2: thrombosis Soluble FGL2: immunomodulation	CD80↓, MHC-II↓ IL-2↓, IFN-γ↓, IL-4↑, IL-10↑ Allogeneic T lymphocyte proliferation↓	(121–123)

	Lactoferrin	Globular glycoprotein (~75−80 kDa). Forms two homologous globular domains. In secretory fluids exists predominantly in tetrameric form	Transfer of iron to the cells. Control of the level of free iron in the blood	DC-SIGN↑, MR↑, CD80↑, CD86↑, and HLA-DR↑, PD-L1↑, ILT3↑ Endocytosis↑ IL-6↑, IL-12↓, TNF↓, IL-23↓ IL-10↓, CCL2↓, CCL1↑ Allogeneic T lymphocyte proliferation↓	(98, 99)

Another key aspect of APP action, as outlined in previous sections, relates to the simultaneous presence and increased levels of both APPs and Mo-DCs under overwhelming immune-inflammatory conditions such as the acute phase response, which incites a physiological crosstalk between these humoral (APPs) and cellular (Mo-DCs) systems with the common goal of providing protection and progress toward the resolution of inflammation. In this regard, APPs probably contribute *in vivo* to mononuclear phagocyte switching toward an anti-inflammatory mode aimed at restoration of tissue integrity and function. Consequently, the APP interaction with Mo-DCs will be safer and effective over a wide range of concentrations, according to the significantly increased blood levels reached by APPs under acute phase conditions. On the other hand, most APPs have been shown to act over a narrow window within the Mo-DC differentiation and/or maturation program, limiting also their hypothetical toxicity, if any, and increasing their specificity compared with some of the current immunosuppressive/immunomodulatory agents, which need to be present over the full differentiation/maturation program to induce a tolerogenic outcome into Mo-DCs. Furthermore, several APPs and, particularly, all soluble PRMs, operate only in the early stages of monocyte to Mo-DC differentiation, while IL-10, for example, is active on Mo-DCs up to their terminal differentiation, when Mo-DCs downregulate the IL-10 receptor (147). This restricted activity of APPs at the beginning of the Mo-DC differentiation program may induce a more permanent and stable modification of the Mo-DC tolerogenic phenotype than that achieved by immunomodulators/immunossupressors influencing Mo-DCs late in their differentiation process, or by agents affecting only their maturation/activation status. These last agents may be more prone to be influenced by the pro-inflammatory microenvironment that the tolerogenic Mo-DCs face upon clinical administration. Comparing the performance and outcome of *in vitro* assays, APPs seem to hold a tolerogenic activity over Mo-DCs (low expression of co-stimulatory molecules, low production of pro-inflammatory cytokines, increased release of anti-inflammatory cytokines, low T cell alloproliferation and, instead, increased Treg generation, etc.) at least as efficient as the agents (rapamycin, dexamethasone and/or vitamin D_3_, NF-κB inhibitors, etc.) currently employed to generate clinical-grade tolerogenic Mo-DCs for the induction or restoration of immune tolerance in autoimmune pathologies and transplantation (148). Furthermore, given the broadened presence that APPs can reach in serum under acute phase conditions and the present hurdles facing adoptive DC-based immunotherapy (time-consuming, expensive, and arduous to implement in the current regulatory environment), the direct *in vivo* administration of APPs, either naked or complexed with nanoparticles, may become a useful and efficacious alternative in inflammatory pathologies. In fact, nanoparticle formulations for DC-specific receptor targeting (DEC205, DC-SIGN, CD40, CD11c, etc.) are being used in preclinical assays and phase I clinical trials as vaccines for onco-immunotherapy (149–151).

Nevertheless, presently the most important drawback for the use of APPs as tolerogenic agents lies in the fact that the detailed molecular mechanisms of action of APP-mediated transformation of Mo-DCs toward a tolerogenic phenotype are not known for most of these proteins. Thus, current efforts employing high-throughput genomics and proteomics approaches will certainly dissect cell surface-interacting partner(s) and relevant signaling and metabolic pathways underlying APP-mediated programming and distinctive functional outcome of the ensuing tolerogenic Mo-DCs (152, 153).

## Conclusion and Prospects

For a successful tolerogenic immunotherapy, Mo-DC conditioning must regulate antigen-specific immune responses in the intrinsically complex pro-inflammatory environments evolving in autoimmune disorders and transplantation, sustaining the development of immunological memory toward tolerance. Thus, it is critically important to thoroughly test the performance of novel tolerance-inducing agents regarding the potency and durability of the ensuing tolerogenic Mo-DC phenotype.

Besides being proposed as useful biomarkers for a variety of inflammatory pathologies, recent studies have proposed that APPs play important roles in tissue homeostasis and repair following overwhelming immune-inflammatory processes, probably in close interaction with inflammatory monocytes and DCs. In fact, APPs are able to generate tolerogenic Mo-DCs *in vitro* with the desired regulatory features (increased expression of immunomodulatory molecules, enhanced production of anti-inflammatory cytokines, and Treg generation) and low immunogenicity (Table 1), comparable with the currently used clinical tolerogenic Mo-DC-inducing immunomodulatory/immunosuppressive agents. Although the precise mechanism of action of tolerogenic Mo-DC skewing induced by most APPs is still unknown, these proteins may prove useful alternatives to overcome the present limitations for a more efficacious, safe, and stable Mo-DC-based tolerogenic immunotherapy. In this regard, attractive attributes of APPs include a physiological basis regarding their interaction with Mo-DCs in the context of the acute phase response, and a wide range of action due to the own intrinsic features of APPs, which would ensure reduced toxicity at the cellular level and increased safety upon *in vivo* administration. Moreover, the narrow activity window in the early stages of monocyte to Mo-DC differentiation shown by several APPs, notably soluble PRMs, should increase specificity and, more importantly, may contribute to a more stable tolerogenic phenotype. APPs targeting differentiating Mo-DCs could turn these cells unresponsive to the *in vivo* pro-inflammatory microenvironment present in autoimmune or alloimmune conditions and, therefore, refractory to Mo-DC maturation. Furthermore, taking into account novel findings, such as the proteomic characterization of tissue-/disease-specific posttranslational modifications of APPs (14), or the influence of the clinical status of the Mo-DC recipient (154) may fine-tune the tolerogenic potential of APP-treated Mo-DCs, e.g., their ability to modulate T cell responses. Nevertheless, additional research should help clarify whether some APPs, particularly pentraxins, or complement activators, are able to maintain their induced tolerogenic DCs in a stable and functional state upon administration in complex pathological tissue contexts, because of the dual protective and pro-inflammatory role played by these multifaceted molecules in physiology.

Definitely, although further work is warranted to establish which method, or perhaps combination of methods, is most suitable to generate tolerogenic Mo-DCs in the clinical setting, APPs may contribute, either on their own, combined with currently employed immunomodulators/immunosuppressants (155), and/or with recently proposed tolerogenic DC boosters such as minocycline (156), or reinforcing the tolerogenic properties of iPSC-derived CD141^+^ DCs holding enhanced capacity for antigen cross-presentation (157), to the design of tailored protocols to induce or re-establish immunological tolerance in different clinical settings including allogeneic transplantation and autoimmune diseases.

## Author Contributions

JA wrote the manuscript. IS and AL added additional insights. The final version was proofread and edited by all authors.

## Conflict of Interest Statement

The authors declare that the research was conducted in the absence of any commercial or financial relationships that could be construed as potential conflict of interest.
